# A PK2/Bv8/PROK2 Antagonist Suppresses Tumorigenic Processes by Inhibiting Angiogenesis in Glioma and Blocking Myeloid Cell Infiltration in Pancreatic Cancer

**DOI:** 10.1371/journal.pone.0054916

**Published:** 2013-01-23

**Authors:** Valerie F. Curtis, Hui Wang, Pengyuan Yang, Roger E. McLendon, Xiaohan Li, Qun-Yong Zhou, Xiao-Fan Wang

**Affiliations:** 1 Department of Pharmacology and Cancer Biology, Duke University, Durham, North Carolina, United States of America; 2 Department of Pathology, Duke University, Durham, North Carolina, United States of America; 3 Department of Pharmacology, University of California Irvine, Irvine, California, United States of America; National Cancer Center, Japan

## Abstract

Infiltration of myeloid cells in the tumor microenvironment is often associated with enhanced angiogenesis and tumor progression, resulting in poor prognosis in many types of cancer. The polypeptide chemokine PK2 (Bv8, PROK2) has been shown to regulate myeloid cell mobilization from the bone marrow, leading to activation of the angiogenic process, as well as accumulation of macrophages and neutrophils in the tumor site. Neutralizing antibodies against PK2 were shown to display potent anti-tumor efficacy, illustrating the potential of PK2-antagonists as therapeutic agents for the treatment of cancer. In this study we demonstrate the anti-tumor activity of a small molecule PK2 antagonist, PKRA7, in the context of glioblastoma and pancreatic cancer xenograft tumor models. For the highly vascularized glioblastoma, PKRA7 was associated with decreased blood vessel density and increased necrotic areas in the tumor mass. Consistent with the anti-angiogenic activity of PKRA7 *in vivo*, this compound effectively reduced PK2-induced microvascular endothelial cell branching *in vitro*. For the poorly vascularized pancreatic cancer, the primary anti-tumor effect of PKRA7 appears to be mediated by the blockage of myeloid cell migration/infiltration. At the molecular level, PKRA7 inhibits PK2-induced expression of certain pro-migratory chemokines and chemokine receptors in macrophages. Combining PKRA7 treatment with standard chemotherapeutic agents resulted in enhanced effects in xenograft models for both types of tumor. Taken together, our results indicate that the anti-tumor activity of PKRA7 can be mediated by two distinct mechanisms that are relevant to the pathological features of the specific type of cancer. This small molecule PK2 antagonist holds the promise to be further developed as an effective agent for combinational cancer therapy.

## Introduction

The complex tumor microenvironment is an important contributor to tumorigenesis. In recent years, increased focus has been placed on targeting the stromal cells in the tumor microenvironment that are responsible for various aspects of the tumorigenic process. Bone marrow-derived myeloid cells, which are precursors to macrophages, neutrophils and myeloid-derived suppressor cells, represent a subpopulation of stromal cells that play important roles during tumor progression [1]. In response to cytokines/chemokines secreted by tumor cells, myeloid cells can be mobilized from the bone marrow and infiltrate into tumor sites where they can promote growth, invasion and angiogenesis to support tumor expansion and metastasis [2]. For instance, CSF3 (colony stimulating factor 3), also known as G-CSF, produced by tumor cells can lead to the differentiation of CD11b^+^Gr1^+^ myeloid cells into neutrophils, macrophages, and dendritic cells, that have been shown to be overproduced in cancer patients and tumor-bearing mice [1], [3]–[4]. At the tumor site, these CD11b^+^Gr1^+^ myeloid cells secrete a variety of factors that can directly contribute to angiogenesis and tumor growth [5]–[6].

Among those factors produced by the CD11b^+^Gr1^+^ myeloid cells is prokineticin 2 (PROK2), referred to as PK2 in this document, also known as Bv8. As one of two members of the prokineticin family, PK2 binds to two highly related G-protein-coupled receptors (GPCRs), PROKR1, referred to as PKR1 and PROKR2, referred to as PKR2, and affects multiple biological processes including nociception, circadian rhythm, gastrointestinal motility, neurogenesis, hematopoiesis and angiogenesis [7]. PK2 production by CD11b^+^Gr1^+^ myeloid cells can lead to the formation of a positive feedback loop, with enhanced differentiation of these myeloid precursor cells into macrophages, as well as increased mobilization of these cells from the bone marrow into the blood stream [8]. These differentiated macrophages can then infiltrate the tumor microenvironment and continue to secrete more PK2, leading to increased proliferation and migration of endothelial cells expressing PKR1 and PKR2, thus contributing to enhanced angiogenesis [8]. In addition to stimulating endothelial cells, PK2 was shown to affect cytokine production in mouse lymphocytes, increasing pro-inflammatory cytokines IL-1B and IL-12 and decreasing anti-inflammatory cytokines IL-4 and IL-10 [9]–[10]. PK2 receptors are also expressed in mouse macrophages and endothelial cells; consequently PK2 can induce migration of these macrophages and affect the formation of capillary-like structures of endothelial cells [10]–[13].

Because of the important roles of PK2 in the creation of a tumor microenvironment favoring tumor growth and progression, PK2 has become a target for the development of novel cancer therapies. A number of studies have convincingly shown that neutralizing antibodies against PK2 could exhibit a potent anti-tumor effect on multiple types of human cancers in mouse models [1], [6], [8]. Those positive results from the proof-of-principle type of experiments have laid the foundation for further development of anti-PK2 agents into therapeutics. In this study, we report our findings on the anti-tumor activity of a synthetic small molecule of PK2 antagonist, PKRA7 which can compete for the binding of PK2 to its receptors PKR1 and PKR2, consequently inhibiting the ability of PK2 to activate downstream pathways [Zhou, manuscript in preparation]. We chose to test PKRA7 in two tumor types, glioblastoma and pancreatic cancer, that have persistently exhibited the worst prognoses among all cancers due to the lack of effective therapy. The two types of cancer display drastically different pathological features. Glioblastoma is highly vascularized and has shown some sensitivity to anti-angiogenic therapy, whereas pancreatic cancer is often poorly vascularized but highly fibrotic with a large portion of the tumor mass consisting of stromal components including infiltrated macrophages [14]–[16]. However, one common feature of both glioblastoma and pancreatic cancer is the involvement of myeloid cells [17]–[20]. We hoped to determine whether PKRA7 could have an impact on tumor growth through its inhibitory effect on myeloid cells. Consistent with this postulation, our results clearly demonstrate that PKRA7 possesses a strong anti-tumor activity for both types of cancers, although through different mechanisms. Furthermore, our study indicates that PKRA7 has the potential to become one component of combination therapies with standard care currently used in the clinic and this compound holds promise for further development for treatment of those cancers that currently have very poor prognosis.

## Results

### PKRA7 Suppresses Tumor Growth in Nude (nu/nu) Mouse Xenograft Model of Glioblastoma through Inhibition of Angiogenesis

Although an anti-PK2 neutralizing antibody was found by previous studies to display anti-tumor activity, we explored the possibility that small molecules can be developed to achieve the same anti-tumor efficacy with lower costs and easier delivery. After biochemically defining the molecular nature of interactions between PK2 and its receptors [21], we have determined the hexapeptide amino acid sequence AVTIGA in the N-terminus of PK2 is completely conversed among mammalian and non-mammalian species and is critical for activating PKR1 and PKR2 [22]. Mutations in this region including an A1M (alanine to methionine) substitution or addition of methionine to the N-terminus displayed strong antagonist activity in the presence of both PKR1 and PKR2 stably expressed on Chinese hamster ovary (CHO) cells [22]. Following this initial discovery, we have synthesized over 200 small molecule compounds that structurally mimic the PK2 N-terminal region mutant peptides and tested their ability to competitively inhibit the activation of PK2 receptors. From this initial screen, we found over 60 water-soluble compounds that exhibit inhibitory effect on PK2-receptor interaction with binding constant below 20 nM (Zhou, manuscript in preparation). We have chosen compound PKRA7 for our experiments because it could potently inhibit PK2 receptors, with IC50 values of 5.0 and 8.2 nM for PKR1 and PKR2, respectively (Figure S1), and, more importantly, it could penetrate the blood-brain barrier, a feature that could be critical for the treatment of glioblastoma.

**Figure 1:**
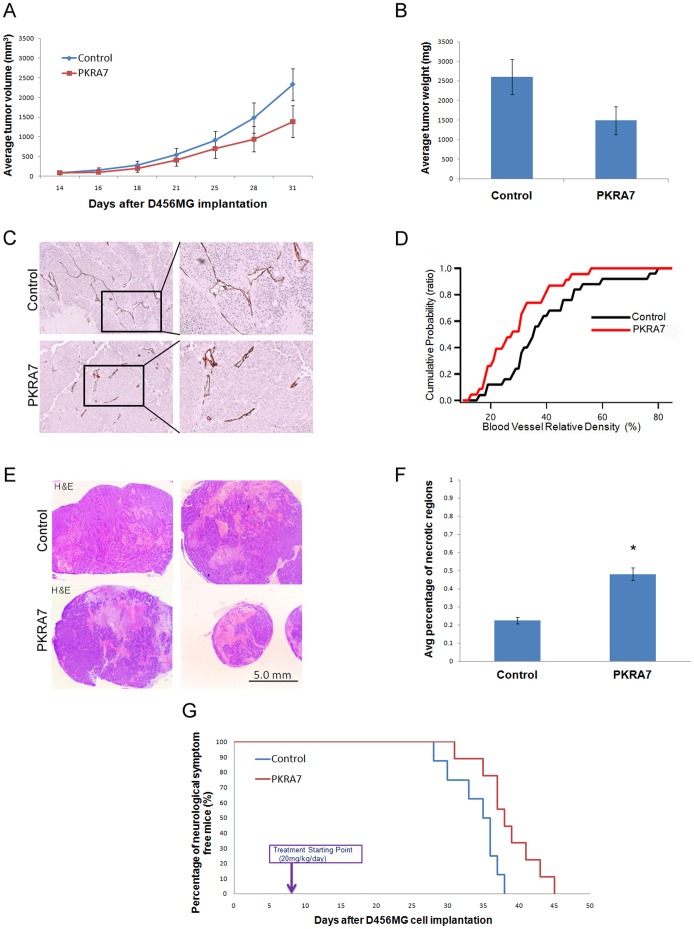
PKRA7 decreases subcutaneous and intracranial glioblastoma xenograft tumor growth. (**A**) D456MG cells were SC injected into nude mice, and control (n = 5) or PKRA7 (n = 5) treatment was commenced when tumors became visually detectable (14 days). Measurements were taken every 2–3 days. (**B**) Average tumor weight of control and PKRA7-treated mouse tumors after removal. (**C**) IHC staining using CD34 endothelial cell marker in D456MG SC tumors from mice treated with control or PKRA7. (**D**) Cumulative probability of vessel relative density as measured by CD34 staining. Vascular density of tumors decreased with PKRA7 treatment. (**E**) Representative pictures of H&E staining of sections from control and PKRA7-treated SC tumors (**F**) Quantification of necrotic regions from 5 slides of each tumor per treatment group, percentages of necrotic areas were measured by ImageJ (*p≤0.05). (**G**) 1×10^4^ D456MG cells were IC injected into nude mice and treatment started 7 days after tumor implantation. Mice in control (n = 8) or PKRA7 treatment (n = 9) group were sacrificed when they developed severe neurological phenotype indicative of tumor growth intracranially.

To study the *in vivo* effect of PKRA7 on glioblastoma tumor growth, we first generated subcutaneous human glioblastoma tumor xenografts in nude mice. 5×10^4^ D456MG glioma cells were implanted into ten nude mice subcutaneously and the mice were separated into two treatment groups 14 days after implantation. The mice in the first group received an intraperitoneal (IP) control treatment of PEG400 (polyethylene glycol) diluted 1∶10 in PBS while the second group of mice received IP injections of PKRA7 in the same solution at a dose of 20 mg/kg/day. Tumor sizes were monitored every three days and growth curves were generated (Figure 1A). 30 days after implantation, the tumors were isolated after the mice were sacrificed and weighed (Figure 1B). Mice treated with PKRA7 showed a clear decrease in both D456MG tumor growth rate and tumor weight. To determine the mechanism by which PKRA7 inhibited xenograft tumor growth, we measured potential changes in blood vessel density and degree of necrosis in D456MG tumors treated or untreated with this compound. As shown in Figure 1C–F, a notable decrease in relative blood vessel density and a significant increase in areas of necrotic regions of the PKRA7-treated tumors were observed in comparison to controls, suggesting that PKRA7 may suppress tumor formation primarily by inhibiting angiogenesis through PKR1 and PKR2 expressed on endothelial cells in a similar fashion as the PK2-neutrolizing antibodies [8], [12]–[13].

Based on these promising results with the suppression of subcutaneous tumor formation by PKRA7, we employed intracranial inoculation of glioma cells to assess the ability of PKRA7 to inhibit tumor growth in a pathologically relevant setting. This time, the treatment started 7 days after 1×10^4^ D456MG glioma cell inoculation with daily IP injections of PKRA7 or vehicle control. Mice were sacrificed when neurological signs of growing tumor burden became evident and the dates were recorded to generate a Kaplan-Meier curve (Figure 1G). In this assay, treatment with PKRA7 noticeably prolonged the onset of neurological signs of tumor burden (mean survival of 38.4 days vs. 34.1 days for PKRA7 and control, respectively, p≤0.05), indicating that PKRA7 was effective in inhibiting tumor growth in the intracranial environment. Similar results were obtained with another glioma cell line as for the D456G cells (data not shown).

### PKRA7 Suppresses Tumor Growth in Nude (nu/nu) Mouse Xenograft Model of Pancreatic Cancer through Inhibition of Macrophage Infiltration

**Figure 2:**
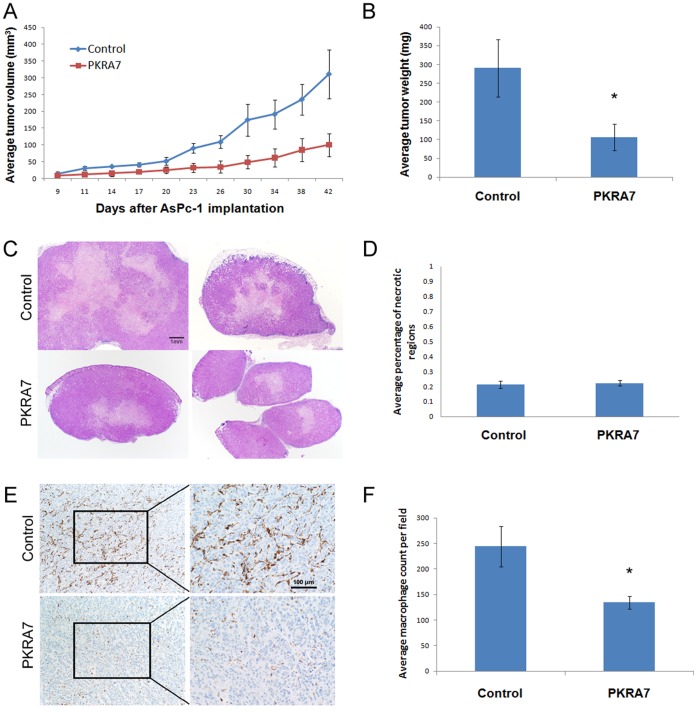
PKRA7 decreases subcutaneous pancreatic cancer xenograft tumor growth. (**A**) AsPc-1 cells were SC injected into nude mice, and control (n = 4) or PKRA7 (n = 5) treatment was commenced when tumors became visually detectable (9 days). Measurements were taken every 2–3 days. (**B**) Average tumor weight of control and PKRA7-treated mouse tumors after removal (*p≤0.05). (**C**) Representative H&E slides from control and PKRA7 treated tumors. (**D**) Quantification of necrotic regions from 5 slides of each tumor per treatment group, percentages of necrotic areas were measured by ImageJ (p = 0.205719). (**E**) IHC staining using F4/80 mouse macrophage marker of AsPc-1 SC tumors treated with control or PKRA7. (**F**) Quantification of average macrophage infiltration of AsPc-1 tumors treated with control (n = 4) or PKRA7 (n = 5), 5 slides of each tumor per treatment group (*p≤0.05).

We next tested whether PKRA7 could have an impact on the xenograft growth of human pancreatic cancer cells due to the well-established role of myeloid cells in the formation of pancreatic cancer. 5×10^5^ AsPc-1 cells were inoculated into nude mice subcutaneously and the treatment started 7 days after implantation following the same procedure as with the D456MG glioma cells. As shown in Figure 2A, growth rate of the AsPc-1 cells was suppressed by PKRA7, resulting in a significant reduction in the average weight of the tumors (Figure 2B). Similar results were obtained when a different human pancreatic cancer cell line, CFPac-1, was used in place of AsPc-1 cells (Figure S2).

To determine the potential mechanism underlying the significant reduction in tumor growth due to PKRA7 treatment, we examined tumor sections for signs of changes in angiogenesis and necrosis. There was no difference in the density of blood vessels though there were fewer vessels per field of view observed compared to the glioblastoma sections (data not shown) and similar levels of necrosis were observed for the tumors derived from treated and control mice (Figure 2C, D). In contrast, there was a significant decrease in the number of macrophages infiltrated into the tumors isolated from PKRA7-treated mice as measured by staining intensity of the mouse macrophage marker F4/80 (Figure 2E, F). These results strongly suggest that PKRA7 could inhibit pancreatic tumor growth via a different mechanism by blocking PKR1- and PKR2-expressing macrophages from infiltrating into the tumor microenvironment rather than suppressing angiogenesis, an observation consistent with the phenotypic features of human pancreatic cancer as poorly vascularized but exhibiting abundant desmoplastic fibrosis and containing large number of infiltrated myeloid cells including macrophages [10], [15].

### PKRA7 Inhibits Endothelial Cell Capillary Branching and Myeloid Cell Migration

**Figure 3:**
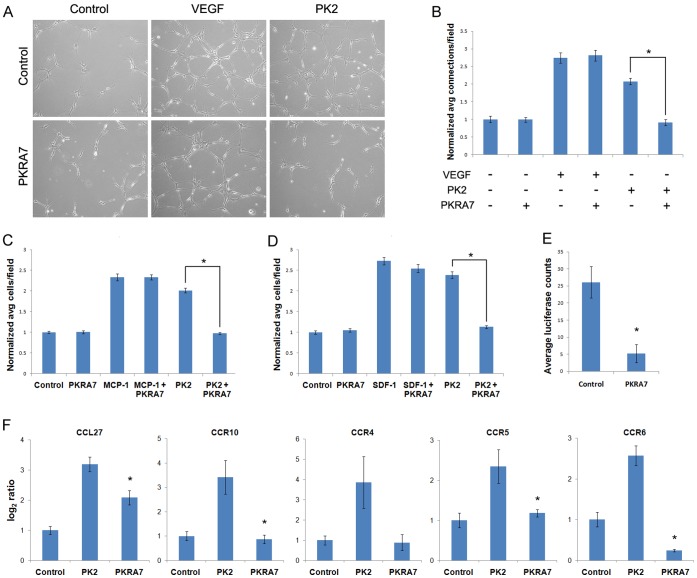
PKRA7 blocks endothelial cell branching, myeloid cell migration, and PK2-induced expression of specific chemokine/chemokine receptors. (**A**) IHMVECs were plated on Matrigel in indicated treatment groups. Representative photographs were taken at 8 hours after plating. (**B**) Average number of connections between cells was counted and analyzed. Results are normalized data from 3 independent experiments. Addition of PKRA7 significantly blocks PK2-induced capillary branching (*p≤0.05). (**C**) 1×10^5^ THP-1 cells on top chamber of transwells were allowed to migrate for 4 hours. Cells were fixed, stained and the number of cells per field of view counted. Results are the normalized average of 3 independent experiments. Addition of PKRA7 significantly blocked PK2-induced monocyte migration (*p≤0.05). (**D**) 7.5×10^4^ RAW264.7 cells on top chamber of transwells were allowed to migrate for 18 hours. Cells were fixed, stained and the number of cells per field of view counted. Results are the normalized average of 3 independent experiments. Addition of PKRA7 significantly blocked PK2-induced macrophage migration (*p≤0.05). (**E**) Average measured luminescence of tumor site after IP injection of luciferase-labeled RAW cells into control (n = 4) or PKRA7 (n = 4) treated mice with SC AsPc-1 tumors 30 days after implantation. Average total luciferase counts were lower in mice treated with PKRA7 compared to control (*p≤0.05). (**F**) qPCR assay to measure the effect of PKRA7 on the expression of chemokines and chemokine receptors that were identified to be induced by PK2 treatment. Data on the mRNA level changes were shown as log2 of the Ct value changes. PKRA7 inhibits upregulation of CCL27, CCR10, CCR4, CCR5, and CCR6 (*p≤0.05).

The results from the *in vivo* xenograft studies of human glioma and pancreatic cancer cells strongly supported the notion that the anti-tumor activity of PKRA7 could be mediated via two very different mechanisms. To probe this further at the cellular level, we conducted *in vitro* assays to assess the impact of PKRA7 on the angiogenic activity of endothelial cells, as well as the migratory ability of myeloid cells. To determine whether PKRA7 affects the ability of endothelial cells to form capillary tube-like network as an important indicator of angiogenesis [23], we employed immortalized human microvascular endothelial cells (IHMVECs). The cells were treated with 200 ng/ml recombinant PK2 alone or PK2 plus 1 µg/ml PKRA7 and plated onto a thin layer of Matrigel. This concentration of PKRA7 was used in our *in vitro* experiments because it is close to the concentration of PKRA7 in the circulation of mice from our *in vivo* experiments while remaining non-toxic to cells in tissue culture (data not shown). As shown in Figure 3A, PKRA7 effectively inhibited PK2-induced capillary branching as measured by the number of connections between cells, but had no effect on VEGFA-induced capillary branching (quantification presented in Figure 3B). Identical results were obtained using two additional endothelial cell lines: mouse embryonic endothelial cells and primary human microvascular endothelial cells (data not shown). The specificity of the anti-PK2 activity in this assay was further confirmed by the demonstration of a similar effect by the anti-PK2 polyclonal antiserum (Figure S3 and data not shown). Taken together, these results suggest that PKRA7 can specifically inhibit the angiogenic effect of PK2 on endothelial cells.

To determine the effect of PKRA7 on PK2-induced migration of myeloid cells, we employed the human monocyte cell line, THP-1 using a transwell migration assay. As shown in Figure 3C, 1 µg/ml PKRA7 effectively impaired the PK2-induced migration of the THP-1 cells, but not the migration of those cells towards CCL2 or monocyte chemotatic protein 1 (MCP-1), a known chemoattractant of THP-1 [24]–[25]. Nearly identical results were observed with the mouse macrophage cell line, RAW264.7 with PKRA7 specifically blocking PK2-induced migration but not migration induced by CXCL12, here referred to as SDF-1α (Figure 3D). Therefore, PKRA7 specifically inhibits PK2-induced migration of myeloid cells from both human and murine origins.

To assess the impact of PKRA7 on migration/infiltration of mouse macrophages into the microenvironment of xenograft tumors formed by human pancreatic cancer cells, we measured accumulation in the tumors of luciferase-labeled RAW264.7 macrophage cells 24 hours following their IP injection into the nude mice 30 days after subcutaneous implantation of AsPc-1 cancer cells. As shown in Figure 3E, a significant decrease in luminescent signal emitted by the mouse macrophage cells was observed in mice treated with PKRA7 in comparison to that of the control mice. These results indicate that PKRA7 is able to block macrophage migration/infiltration into the tumor site in an *in vivo* setting, thus inhibiting the ability of the macrophages to positively contribute to the growth of xenograft tumors.

To further examine the mechanism by which PKRA7 blocks PK2-induced macrophage migration, we performed a cytokine array using quantitative-real time PCR on THP-1 cells that were induced to differentiate into macrophage cells. Among an array of 95 human chemokine/cytokine ligands and their receptors, five displayed a significant induction in their expression after treatment with PK2 including CCL27, CCR10, CCR4, CCR5, and CCR6 (Figure S4). Importantly, at least four of these induced molecules are known to be involved in enhancing the migration of myeloid cells and all of their induction by PK2 was blunted by PKRA7 (Figure 3F), strongly suggesting that suppression of the PK2-induced production of these chemokines and receptors underlies the primary mechanism of anti-tumor activity of PKRA7 in the context of pancreatic cancer.

### PKRA7 Enhances the Efficacy of Standard Therapies for Glioblastoma and Pancreatic Cancer in Xenograft Models

**Figure 4:**
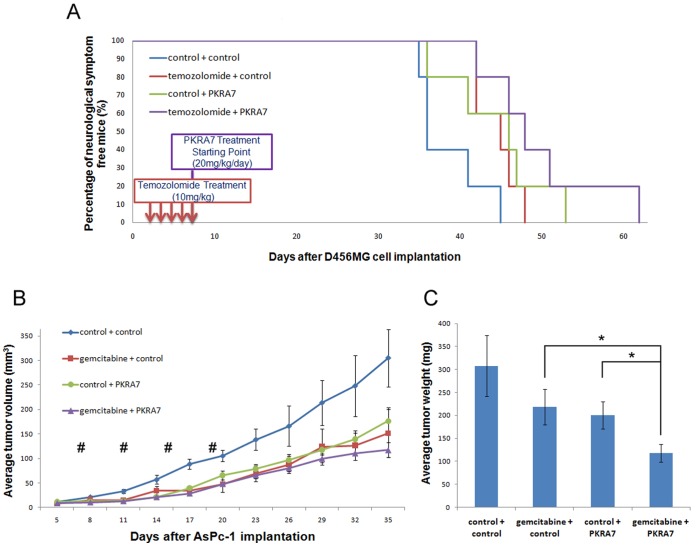
PKRA7 enhances the efficacy of chemotherapeutic drugs to reduce glioblastoma and pancreatic xenograft tumor growth. (**A**) Kaplan-Meier curve of nude mice after temozolomide and PKRA7 treatment following IC injection of 1×10^4^ D456MG cells. Treatment with 10 mg/kg temozolomide or control started 3 days after IC injection for a total of 5 consecutive daily treatments. Treatment with PKRA7 or control started 7 days after IC injection and continued for the duration of experiment. 5 mice per condition. (**B**) AsPc-1 cells were SC injected into nude mice, and control (n = 10) or PKRA7 (n = 10) treatment was commenced when tumors were visible (7 days). Treatment with 100 mg/kg gemcitabine (n = 10) or control (n = 10) started 7 days after tumor implantation and was administered every 4 days for two weeks for a total of 4 treatments (#). Measurements were taken every 3 days. 5 mice per condition. (**C**) Average tumor weight of control, gemcitabine, PKRA7 and gemcitabine plus PKRA7-treated mouse tumors after their removal (*p≤0.05).

Although PKRA7 displayed strong anti-tumor activities in the contexts of both glioblastoma and pancreatic cancer, it is unlikely to be developed into a therapeutic agent used alone. To test whether PKRA7 could increase the efficacy of standard chemotherapeutic treatment for glioblastoma, we examined the effect of this compound in combination with temozolomide that is currently used in the clinic for this disease [26]–[27]. Following an established experimental procedure for evaluating the effect of combinational therapy in xenograft mouse models [28]–[29], 1×10^4^ D456MG cells were implanted intracranially and followed by treatment with 10 mg/kg temozolomide for five days, and then with or without PKRA7 administration during the remaining days of the experiments. As shown in Figure 4A, treatment with both temozolomide and PKRA7 prolonged the onset of neurological signs of tumor burden compared to mice receiving control, temozolomide or PKRA7 alone, indicating an enhanced effect of combinational therapy with the agents in inhibiting intracranial glioma growth in nude mice (mean survival of 49.8 days for PKRA7 plus temozolomide vs 44.6 days for either temozolomide or PKRA7 alone vs 38.6 days for control).

For pancreatic cancer, gemcitabine is one of the main chemotherapeutic drugs currently used in the clinic and it was tested previously in combination therapy studies involving AsPc-1 cells [30]. In our experiments, 5×10^5^ AsPc-1 cells were subcutaneously implanted into nude mice that were treated with PKRA7 and gemcitabine (100 mg/kg) 7 days later. As shown in Figure 4B, it is clear that tumors from mice received both gemcitabine and PKRA7 were significantly smaller than those from mice treated with only gemcitabine or PKRA7. These results suggest that combinational therapy with gemcitabine and PKRA7 could have a stronger anti-tumor effect than the standard therapy to reduce pancreatic cancer xenograft tumor growth in our model.

## Discussion

Tumorigenesis is a complex process that involves much more than proliferating tumor cells. The tumor cells are aided and supported by the surrounding tumor stromal microenvironment that is rich with a heterogeneous mix of cells such as fibroblasts, endothelial cells and immune cells [31]. These cells respond to signals from the expanding tumor by secreting factors of their own that can perpetuate the growth signal, remodel the extracellular matrix, contribute to angiogenesis and redirect the role of immune cells. Angiogenesis has long been understood to be an important part of tumorigenesis and many studies have been done to block this process [32]. While important angiogenic factors have been identified such as VEGF, therapeutics against the VEGF signaling pathway have not been proven to be universally successful. Indeed, many cancers are resistant to anti-VEGF therapy or become refractory to administration of these therapies, resulting in recurrence that is sometimes more aggressive than the primary tumor [14], [16], [33]–[38].

There is a need to identify and target additional signaling pathways that may contribute to angiogenesis or other processes that have been shown to be important for tumor progression such as macrophage infiltration. PK2 and its receptors are part of a signaling pathway involved in myeloid cell mobilization [8]. Multiple studies have shown that the CD11b^+^Gr1^+^ myeloid precursor cells can contribute to angiogenesis and tumorigenesis in a variety of cancer types [8], [37], [39]. Macrophages derived from those precursor cells in the tumor microenvironment can also secrete cytokines that directly affect tumor cell growth. In recent studies, the anti-tumor efficacy of an anti-PK2 antibody has been compared to treatment with an anti-VEGF antibody and found to be nearly as effective in preventing disease progression of a transgenic mouse model of pancreatic β-cell tumorigenesis, while the combination of the two antibodies showed an even more pronounced effect in inhibiting subcutaneous growth of different human cancer cell lines (colon cancer, rhabdomyosarcoma) and mouse tumor cells (mastocytoma, lymphoma) [8], [39]. Anti-PK2 antibody treatment also reduced the number of circulating and tumor-infiltrating CD11b^+^Gr1^+^ myeloid cells, including bone marrow-derived macrophages, which have been shown to mediate refractoriness to anti-VEGF therapies in several mouse xenograft tumor models [8], [37]. Those studies have indicated PK2 as a legitimate target for cancer therapy.

Antibody-based therapies represent a significant portion of cancer treatment options in today’s clinics. However, studies with patients and mouse models of glioblastoma and pancreatic cancer have shown that these types of cancer can be resistant or refractory to anti-VEGF signaling therapies [14], [16], [33], [35]–[37]. Other studies have shown that this resistant response may be common to additional types of cancer such as breast and colon cancer [34], [40]–[41]. Patients may therefore benefit from additional therapies that target alternate pathways in combination with anti-VEGF signaling therapies to prevent refractory responses. Thus, small molecule inhibitors offer an alternative therapeutic approach because they can still be specific to their targets while being more cost effective to manufacture. Also, some drug therapies are required to cross blood-brain barrier to treat diseases such as glioblastoma and small molecule inhibitors are good candidates for this purpose. In this regard, our demonstration that PKRA7 is capable of penetrating the blood-brain barrier in the mouse and acts to inhibit intracranial xenograft tumor formation by glioma cells presents an alternative strategy to inhibit tumor angiogenesis via a mechanism distinct from that of anti-VEGF since PK2 enhances angiogenesis through its G-protein coupled receptor activated pathways [7].

Desmoplastic stroma is a defining feature of pancreatic cancer and can contain high levels of tumor-associated macrophages (TAMs), especially at the invasive front of pancreatic cancer [15], [18]. This infiltration of macrophages is thought to contribute to disease progression and is associated with poor prognosis [18]. Recent studies report that immunosuppressive cells including TAMs, myeloid-derived suppressor cells (MDSCs) and regulatory T cells (T_reg_) were found in high levels in early to late stages of progressing cancer compared to normal tissue in a mouse model of pre-invasive and invasive pancreatic ductal adenocarcinoma [42]. PK2 may play a critical role in the complex and dynamic relationship between pancreatic cancer cells and these stromal cells through the regulation of recruitment and activity of myeloid cells. Indeed, we have found that PK2 induces the production of chemokines and receptors involved in the migration of myeloid cells and PKRA7 could block this induction (Figure 3F). The chemokine receptors, CCR10 and CCR4, known to respond to chemo-attractants CCL27, MCP-1, and CCL22, have been shown to be critical in inducing mobilization and homing of myeloid cells and leukocytes to the tumor site [43]. The chemokine receptor CCR6 and its ligand CCL20 have been implicated in dendritic cell migration and appear to be important for maintaining a normal level of the macrophage population since CCR6^−/−^ mice showed decreased numbers of macrophage cells [44]. Our studies showed that pancreatic tumors are poorly vascularized and contain relatively few endothelial cells in the untreated mice and confirmed that any changes in vascular density due to PKR inhibition by PKRA7 in the treated mice would likely be very small and difficult to detect. Taken together with data showing the response of myeloid cells to PK2, it is clear that PKRA7 acts to suppress pancreatic cancer by blocking the ability of PK2 to induce myeloid cells mobilization from the bone marrow specifically as well as PK2’s direct effect on the tumor microenvironment through pro-migratory responses.

Combining PKRA7 with the established chemotherapeutic treatments temozolomide and gemcitabine resulted in enhanced effects in our glioblastoma and pancreatic cancer xenograft models, respectively, fully demonstrating the potential of developing this compound as a component of combinational therapies. A multi-pronged approach to cancer treatment is the most effective way to combat this deadly disease. Cancers that have been identified to be refractory or resistant to certain treatments may require multiple therapeutic options to more effectively block the pathways involved in tumorigenesis. For example, patients with glioblastoma may benefit from treatments blocking both VEGF and PK2 signaling pathways in combination with a chemotherapeutic therapy. Ongoing research in this nature will continue to lead to identification of new pathways and targets, such as PK2, for the development of more personalized treatment options for cancer patients.

## Materials and Methods

### Ethics Statement

The Duke University Institutional Animal Care and Use Committee approved this study (protocol A189-11-07 (1)). All studies were performed in accordance with the Institute of Laboratory Animal Research (NIH, Bethesda, MD) Guide for the care and Use of Laboratory Animals.

### PKRA Synthesis

PKRA7 was synthesized to mimic mutated peptides of the N-terminal region of PK2. Mutated peptides of this region including an alanine to methionine (A1M) substitution and addition of a methionine to the N-terminus were created. Small molecule PKR antagonists were chemically synthesized to mimic these inhibitory peptide mutants and PKRA7 was chosen for further studies due to its low IC50 values. A detailed description of the synthesis of PKRA7 can be found in the supporting information (Method S1. Detailed synthesis of PKRA7).

### Cell Culture

The following cells were cultured in the indicated media (and grown at 37°C, 5% CO_2_): D456MG: Neurobasal (Invitrogen, Carlsbad, CA) supplemented and maintained as described [45]; AsPc-1 (ATCC), CFPac-1 (ATCC) and THP-1 (ATCC): RPMI 1640 (Mediatech, Inc, Manassas, VA), 10% fetal bovine serum (FBS; Invitrogen), Penicillin/Streptomycin Solution (P/S, Mediatech, Inc); RAW264.7 (ATCC): DMEM (Mediatech, Inc), 10% FBS, P/S; immortalized HMVEC [46]: EBM-2 (Lonza, Basel, Switzerland), EGM-2 MV SingleQuots (Lonza), P/S; mouse embryonic endothelial cells (MEEC) [47]: MCDB-131 (Invitrogen) supplemented with 15% FBS, 2 mM L-glutamine, 1 mM sodium pyruvate (Invitrogen), 100 µg/mL of heparin (Sigma-Aldrich, St. Louis, MO), and 50 µg/ml endothelial cell growth supplement (ECGS) (Sigma-Aldrich); human microvascular endothelial cells (HMEC-1) [47]: MCDB-131 medium (Invitrogen), supplemented with 10% FBS, 1µg/ml hydrocortisone (Sigma), 10 ng/ml EGF (Sigma) and 2 mM L-glutamine (Invitrogen).

### Xenograft Assays

Athymic *nu/nu* mice were maintained in HEPA-filtered facilities in the Duke University Cancer Center Isolation Facility. Intracranial (IC) or subcutaneous (SC) transplantations of D456MG, AsPc-1 and CFPac-1 cells into these mice were performed as described [48]–[49]. Briefly, for IC transplantations, 1×10^4^ D456MG cells were implanted into the subventricular zone of the brains of 4–6 week old mice using a 28G1/2″ insulin syringe (Becton Dickinson, Franklin Lakes, NJ). Mice were maintained until the development of neurological symptoms. For SC injections 5×10^4^ D456MG, 5×10^5^ AsPc-1 or 5×10^5^ CFPac-1 cells were implanted subcutaneously on the right flank of nude mice in a volume of 50µl using previously mentioned insulin syringes. SC tumors were measured with hand-held vernier calipers (Bel-Art Products, Pequannock, NJ) and tumor volume was calculated based on the following formula: [(π/6) x (width)^2^ x (length)]. In all animal experiments, animals were treated with 20 mg/kg PKRA7 or phosphate-buffered saline injected intraperotineally with previously mentioned insulin syringes every day until the termination of experiments, at which time IC injected mice were sacrificed and brains were harvested and examined or SC injected mice were sacrificed and tumors were harvested, weighed, and examined. In combination experiments, as well as daily injections of 20 mg/kg PKRA7, 10 mg/kg of temozolomide was administered to mice receiving D456MG IC injections 3 days after injection and received 5 consecutive treatments. 100 mg/kg gemcitabine (Eli Lilly, Indianapolis, IN) was administered to AsPc-1 SC injected mice 1 week after injection and received 4 total injections for 2 weeks (4 days apart).

### Immunohistochemistry and Analysis

Tumors collected from sacrificed mice were embedded in paraffin and stained with H&E or desired antibodies for IHC analysis and counterstained with hematoxylin.

For necrotic areas, 5 slides per tumor (both D456MG and AsPc-1) were H&E stained. High resolution images were taken of each slide at 1× magnification and these slides were analyzed using the ImageJ program. For each slide, the area of the total tumor section was measured in ImageJ. Then, areas identified histologically as necrotic were also measured in ImageJ. The ratio of necrotic area per tumor section over the total tumor section was calculated for each tumor section. These calculations were averaged in two groups: mice receiving control treatment and those receiving PKRA7 treatment.

For measuring CD34 positive cells, 5 slides per tumor (both D456MG and AsPc-1) were stained by mouse CD34 antibody (Abcam, Cambridge, UK). Five images were taken at 20x of each slide (total 25 fields for each tumor) and these images were analyzed using the ImageJ program. For each field, the area of CD34-positive shown in brown was measured in ImageJ. Then, the ratios of CD34 positive area compared to the area of whole field were calculated and analyzed.

For measuring F4/80 positive cells, 1 slide per AsPc-1 subcutaneous tumor was stained by the murine macrophage marker, F4/80 antibody (AbD Serotec, Oxford, UK). High resolution images were taken at 10x, with 3 to 4 images taken per tumor section, depending on its size. The number of positively stained cells was counted on each image. These numbers were averaged as macrophage count per field for mice receiving control treatment and those receiving PKRA7 treatment.

### Capillary Branching Assay

Twelve-well plates were coated with 200 µl Matrigel Matrix (BD Biosciences, San Jose, CA) and allowed to solidify at 37°C. For IHMVEC cells, 3×10^4^ cells were plated in 1 ml EBM-2 media with the following treatment conditions: untreated, VEGF^165^ (100 ng/ml; PeproTech, Rocky Hill, NJ), 200 ng/ml PK2 (200 ng/ml; PeproTech), PKRA7 (1 µg/ml), VEGF^165^+ PKRA7, or PK2+ PKRA7 in triplicate per condition. VEGF and PK2 were dissolved in water plus 0.1% BSA (water +0.1%BSA used as control). Control, PK2, PK2+ PKRA7 or PK2+1% B6246 (anti-PK2 serum) were conditions used for IHMVEC cells in additional experiments. Three images were recorded of each well at each time point. The number of connections between cells was counted, averaged and normalized.

### Transwell Migration Assay

The bottom chambers of 24-well transwell plates (8 µm pore polycarbonate membrane transwell; Corning, Corning, NY) contained 600 µl of each assaying condition in complete media (RPMI +10% FBS, P/S for THP-1 cells) or minimal media (DMEM for RAW264.7 cells). For THP-1 cells the bottom chambers of the transwells containing RPMI media were untreated or contained PKRA7 (1 µg/ml), MCP-1 (100 ng/ml; PeproTech), MCP-1+ PKRA7, PK2 (200 ng/ml; PeproTech), or PK2+ PKRA7 in triplicate per condition. For RAW264.7 cells the bottom chambers of the transwells containing DMEM media were untreated or contained PKRA7 (1 µg/ml), SDF-1α (200 ng/ml; PeproTech), SDF-1α+PKRA7, PK2 (200 ng/ml), or PK2+ PKRA7 in triplicate per condition. MCP-1, SDF-1α and PK2 all dissolved in water plus 0.1% BSA (water +0.1% BSA used as control). The appropriate number of cells was collected in complete (THP-1) or minimal media (RAW264.7) and 100 µl were plated onto the top chamber. The transwell plates containing the cells were placed in an incubator and the cells were allowed to migrate for 6 hours (THP-1) or 18 hours (RAW264.7). The cells were then fixed with 4% PFA, stained with 0.5% toluidine blue in 4% PFA, counted using a microscope and analyzed.

### qPCR Cytokine Array

Cell culture: THP-1 cells were cultured as described [50]–[51]. To induce differentiation, cells were incubated with 10 ng/ml PMA (Sigma-Aldrich) for three days in 35-mm tissue culture dishes. The cells were washed with PBS to remove the PMA and then cultured in PMA-free media another 1 or 2 days. Then, recombined human PK2 protein (200 ng/mL) was added into the medium for 4 hrs. To examine effect of PKRA7 on chemokine expression, the THP-1 macrophages were pre-treated with 1 µg/mlPKRA7 for 0.5 h, then treated with PK2 for 4 h.

qPCR array: Prior to amplification of cDNA, total RNA was isolated using TRIZOL reagent (Bethesda Research Labs, Gaithersburg, MD) according to the manufacturer’s procedures. This is a modification of the acid-guanidinium-phenol extraction method of Chomczynski (1993). The concentration of RNA in any sample was measured by spectrophotometry. Samples were stored at −70°C until used for reverse transcription. Reverse transcription (RT) was carried out using SuperScript® III Reverse Transcriptase (Invitrogen). qPCR-based array for detection of cytokines, chemokines and their receptors was achieved with gene-specific primers. All the mRNA levels (ΔCt) were normalized to β-actin. Data of the mRNA level changes were shown as log2 of the Ct value changes (ΔΔCt = ΔCt_PK2-treated_−ΔCt_Ctrl_).

### In vivo Migration Assay

Subcutaneous tumors were grown in 8 nude mice as previously described, 4 mice receiving daily 20 mg/kg PKRA7 IP injections, 4 receiving control injections. 30 days after cell inoculation, 5×10^5^ luciferase-labeled RAW264.7 cells were IP injected into each mouse. 24 hours later mice were sedated with Ketamine plus Xylazine (100 mg/kg plus 5–10 mg/kg IP), IP injected with the luciferase substrate and imaged using the Xenogen Imager. Photos were taken and luciferase signal at site of tumor was measured and analyzed.

### Statistical Analysis

Result values were expressed as means and SEM and significance was established by one-way ANOVA. In all analyses, the level of statistical significance was 95% confidence level (p, 0.05) and * means p≤0.05.

## Supporting Information

Figure S1 **Potency of PKRA7 in antagonizing PKR1 and PKR2.** Antagonist potency was examined in Chinese hamster ovary (CHO) cells that stably express PKR1 or PKR2. Inhibition of PKR1 or PKR2 activation by PK2/PK2 in the presence of different concentrations of PKRA7 was measured with a luminometer. RLU is an index for calcium influx measurement for this luminescence-based assay. The IC50 of PKRA7 for PKR1 and PKR2 were determined to be 5.0 and 8.2 nM, respectively.
(TIF)

Figure S2 **PKRA7 decreases subcutaneous xenograft tumor growth of another pancreatic cancer cell line.** (**A**) CFPac-1 cells were SC injected into nude mice, and control or PKRA7 treatment was commenced when tumors were visible (6 days). Measurements were taken every 2–3 days (**B**) Average tumor weight of control and PKRA7-treated mice after tumor removal (*p≤0.05).
(TIF)

Figure S3 **PK2-induced branching of endothelial cells was blocked by anti-PK2 anti-serum.** IHMVECs plated on Matrigel were untreated or treated with 200 ng/ml PK2, 1% B6246 anti-PK2 serum, or PK2+ B6246 anti-PK2 serum. Representative photographs were taken at 4 hours after plating and analyzed (*p≤0.05).
(TIF)

Figure S4 **Representative results from the cytokine/chemokine array analysis.** qPCR-based array for detection of cytokines, chemokines and their receptors was achieved with gene-specific primers using THP-1 macrophages treated or untreated with PK2 for 4 h. All the mRNA levels (ΔCt) were normalized to β-actin. Data of the mRNA level changes were shown as ΔΔCt = ΔCt_PK2-treated_−ΔCt_Ctrl_. Box region represents most highly upregulated genes that were used in further studies with PKRA7 as shown in Figure 3F.
(TIF)

Method S1 **Detailed synthesis of PKRA7.**
(DOC)
