# Supplementary figures and images for: A PK2/Bv8/PROK2 Antagonist Suppresses Tumorigenic Processes by Inhibiting Angiogenesis in Glioma and Blocking Myeloid Cell Infiltration in Pancreatic Cancer
Source: PLoS One. 2013 Jan 23;8(1):e54916. doi: 10.1371/journal.pone.0054916 (PMC3553000; doi:10.1371/journal.pone.0054916)

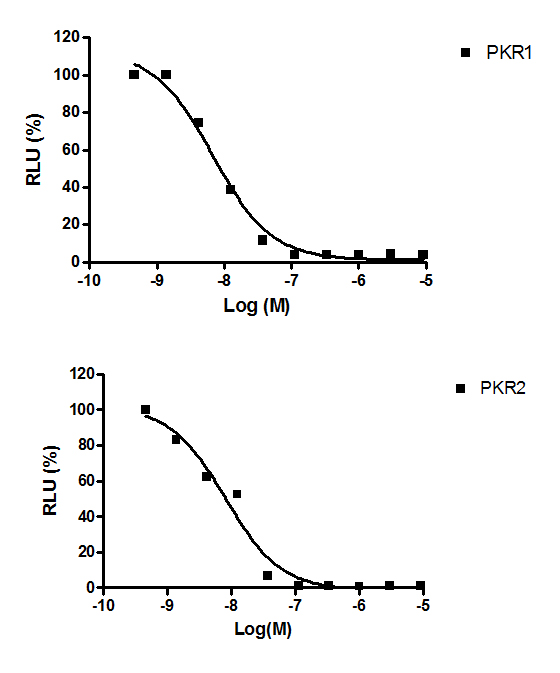

Supplement: Figure S1 — Potency of PKRA7 in antagonizing PKR1 and PKR2. Antagonist potency was examined in Chinese hamster ovary (CHO) cells that stably express PKR1 or PKR2. Inhibition of PKR1 or PKR2 activation by PK2/PK2 in the presence of different concentrations of PKRA7 was measured with a luminometer. RLU is an index for calcium influx measurement for this luminescence-based assay. The IC50 of PKRA7 for PKR1 and PKR2 were determined to be 5.0 and 8.2 nM, respectively. (TIF) [file pone.0054916.s001.tif]

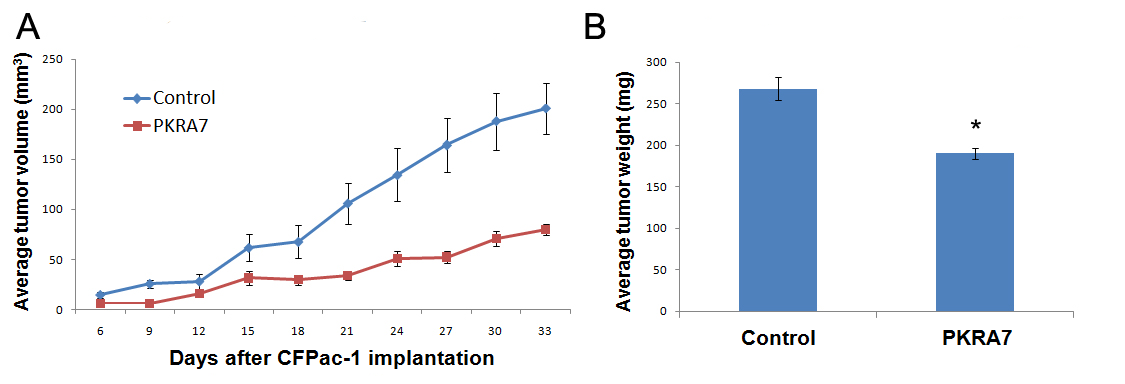

Supplement: Figure S2 — PKRA7 decreases subcutaneous xenograft tumor growth of another pancreatic cancer cell line. (A) CFPac-1 cells were SC injected into nude mice, and control or PKRA7 treatment was commenced when tumors were visible (6 days). Measurements were taken every 2–3 days (B) Average tumor weight of control and PKRA7-treated mice after tumor removal (*p≤0.05). (TIF) [file pone.0054916.s002.tif]

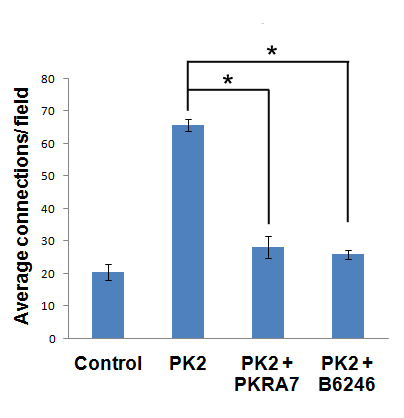

Supplement: Figure S3 — PK2-induced branching of endothelial cells was blocked by anti-PK2 anti-serum. IHMVECs plated on Matrigel were untreated or treated with 200 ng/ml PK2, 1% B6246 anti-PK2 serum, or PK2+ B6246 anti-PK2 serum. Representative photographs were taken at 4 hours after plating and analyzed (*p≤0.05). (TIF) [file pone.0054916.s003.tif]

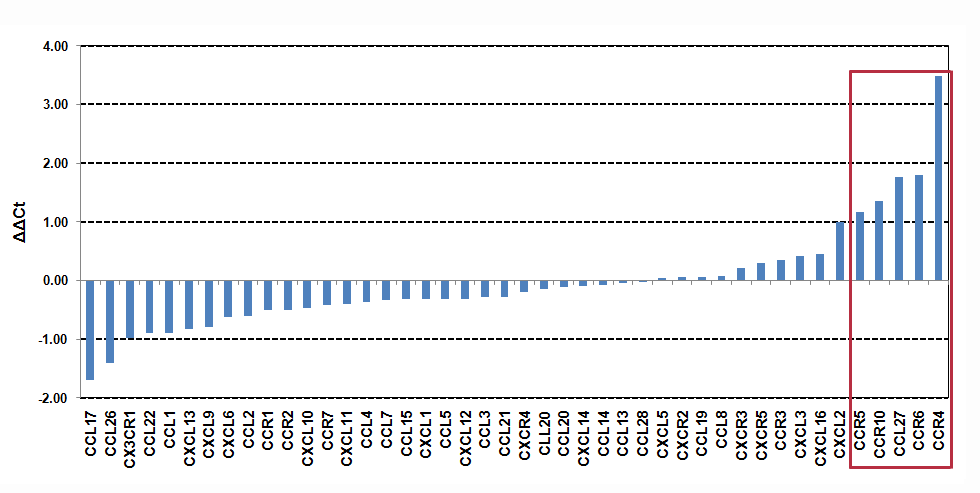

Supplement: Figure S4 — Representative results from the cytokine/chemokine array analysis. qPCR-based array for detection of cytokines, chemokines and their receptors was achieved with gene-specific primers using THP-1 macrophages treated or untreated with PK2 for 4 h. All the mRNA levels (ΔCt) were normalized to β-actin. Data of the mRNA level changes were shown as ΔΔCt = ΔCtPK2-treated−ΔCtCtrl. Box region represents most highly upregulated genes that were used in further studies with PKRA7 as shown in Figure 3F. (TIF) [file pone.0054916.s004.tif]
