# Supplementary material for: A PK2/Bv8/PROK2 Antagonist Suppresses Tumorigenic Processes by Inhibiting Angiogenesis in Glioma and Blocking Myeloid Cell Infiltration in Pancreatic Cancer
Source: PLoS One. 2013 Jan 23;8(1):e54916. doi: 10.1371/journal.pone.0054916 (PMC3553000; doi:10.1371/journal.pone.0054916)
Supplement: Method S1 — Detailed synthesis of PKRA7. (DOC) [file pone.0054916.s005.doc]

Detailed Synthesis of PKRA7

PKRA7 [(3*R*)-1-(4-Fluoro-3-methoxybenzyl)-N-(9-chloro-3,4-dihydro-2H-1,5-benzodioxepin-7-ylmethyl)-N-isobutylpyrrolidine-3-carboxamide] was synthesized as follows:

Step1: A solution of 3-chloro-4-hydroxy-5-methoxy benzaldehyde in dichloromethane was cooled in an ice water bath. Boron tribromide in dichloromethane were added slowly and the mixture was stirred for several hours at room temperature. The mixture was concentrated under reduced pressure. The residue was cooled again with an ice water bath and precipitated with ice-cold aqueous hydrochloric acid. Solid residue was received upon filtration, which was dried to obtain 3-chloro-4,5-dihydroxy benzaldehyde.

Step 2: A mixture of 3-chloro-4,5-dihydroxy benzaldehyde, 1,3-dibromopropane and potassium carbonate was resuspended in acetonitrile and refluxed at 60°C overnight. The mixture was allowed to cool to room temperature and filtered. The filtrate was concentrated under reduced pressure. The residue was purified by flash chromatography on silica (20% ethyl acetate in hexanes) to give the product 9-chloro-3,4-dihydro-2H-1,5-benzodioxepine-7-carbaldehyde as a white crystal.

Step 3: 9-chloro-3,4-dihydro-2H-1,5-benzodioxepine-7-carbaldehyde and isobutylamine were combined in 1,2-dichloroethane and cooled in an ice water bath. Acetic acid was then added, followed by sodium triacetoxyborohydride. The mixture was allowed to stir at room temperature overnight. The reaction mixture was partitioned with aqueous potassium carbonate. The aqueous layer was extracted with dichloromethane. The organic layers were combined, washed with brine, dried over sodium sulfate, and concentrated under reduced pressure. The residue was purified by flash column chromatography (10-100% ethyl acetate in hexanes) to yield N-(9-chloro-3,4-dihydro-2H-1,5-benzodioxepin-7-ylmethyl)-2-methylpropan-1-amine as a light yellow oil.

Step 4: A mixture of N-(9-chloro-3,4-dihydro-2H-1,5-benzodioxepin-7-ylmethyl)-2-methylpropan-1-amine, (R)-Pyrrolidine-1,3-dicarboxylic acid 1-tert-butyl ester and 1-ethyl-3-(3-dimethylaminopropyl)carbodiimide were mixed in dichloromethane and stirred at room temperature overnight. After addition of water, the mixture was extracted with ethyl acetate. The organic layer was washed with saturated sodium bicarbonate, water and brine, dried over anhydrous sodium sulfate and concentrated under reduced pressure. The product [(3*R*)-1-tert-butoxycarbonyl-N-(9-chloro-3,4-dihydro-2H-1,5-benzodioxepin-7-ylmethyl)-N-isobutyl pyrrolidine-3-carboxamide] was purified by column chromatography on silica (33-50% ethyl acetate in hexane) as an oil.

Step 5: The above product was dissolved in dichloromethane, trifluoroacetic acid was then added slowly. The mixture was stirred for several hours at room temperature. After the evaporation of the volatiles under reduced pressure, the residue was partitioned between dichloromethane and a solution of sodium bicarbonate. The aqueous layer was then extracted with dichloromethane. The organic layer was combined, dried with anhydrous sodium sulfate and concentrated under reduced pressure to yield product [(3*R*)-N-(9-chloro-3,4-dihydro-2H-1,5-benzodioxepin-7-ylmethyl)-N-isobutyl pyrrolidine-3-carboxamide] as a lightly reddish oil.

Step 6: A mixture of (3*R*)-N-(9-chloro-3,4-dihydro-2H-1,5-benzodioxepin-7-ylmethyl)-N-isobutyl pyrrolidine-3-carboxamide, 4-fluoro-3-methoxybenzaladehyde, glacial acetic acid, and sodium triacetoxyborohydride were mixed in dichloromethane and stirred overnight. Upon addition of aqueous potassium carbonate, the mixture was extracted with ethyl acetate. The organic layers were combined, washed with brine and concentrated under reduced pressure. Flash chromatography on silica (33-100% ethyl acetate in hexane) yielded the product [(3*R*)-1-(4-Fluoro-3-methoxybenzyl)-N-(9-chloro-3,4-dihydro-2H-1,5-benzodioxepin-7-ylmethyl)-N-isobutylpyrrolidine-3-carboxamide] as a resin. The purity of PKRA was verified by proton NMR and was at least 97%.
